# Twice Daily Prazosin and Valproic Acid in the Treatment of Flashbacks in PTSD

**DOI:** 10.1155/2022/1223292

**Published:** 2022-08-03

**Authors:** Kristy A. Fisher, Heidi Kiziah, Clara L. Villalba Alvarez

**Affiliations:** ^1^HCA Aventura Hospital and Medical Center, USA; ^2^Nova Southeastern University, Dr. Kiran C. Patel College of Allopathic Medicine, USA

## Abstract

Posttraumatic stress disorder (PTSD) is a debilitating stress disorder occurring in the context of a traumatic event and is characterized by intrusive and avoidance symptoms, negative alterations in cognition and mood, and arousal and reactivity changes. Despite its representation throughout literature, the pathophysiology of PTSD remains incompletely understood, thus contributing to broad, variable, and at times, experimental treatment options. The authors present the first documented case of the rapid and successful management of PTSD using valproic acid and twice daily dosing of prazosin aimed at targeting symptoms of hyperarousal and both daily and nightly intrusive symptoms of flashbacks and nightmares, respectively. The authors also discuss postulations of the underlying mechanisms of action responsible for such symptom alleviation. Further investigation is needed to expand upon our knowledge of the use of such agents in the treatment of PTSD to improve upon existing clinical guidelines, especially in the acute setting, thus providing better overall prognosis.

## 1. Background/Introduction

PTSD is a very debilitating disorder with a lifetime prevalence of 3.9% in the general population and 5.6% among those who have endured a traumatic event according to the World Health Organization World Mental Health Surveys which included 26 populations [1]. Research shows even higher prevalence among United States combat veterans, with a 2- to 4-fold increase compared to nonveterans [2]. The *Diagnostic and Statistical Manual of Mental Disorders*, 5^th^ edition (DSM-5) criteria for PTSD include exposure to a traumatic stressor, along with intrusive and avoidance symptoms, as well as negative alterations in cognition and mood, which must last for a duration of at least 1 month. Significant functional impairment must also be evident and other causative disorders and/or medications of such symptoms must be excluded [3]. The treatment algorithm typically includes psychotherapy along with the use of psychotropic medications. The psychotherapy options that have strong recommendations based on recent literature reviews include variations of exposure therapy, cognitive processing therapy, and/or eye movement desensitization and reprocessing (EMDR) [4]. Recommended medications include a selective serotonin reuptake inhibitor (SSRI) or selective norepinephrine reuptake inhibitors (SNRI), such as sertraline, fluoxetine, and venlafaxine [4]. Prazosin has been used for the alleviation of nightmares, although it has recently been the subject of debate. This debate followed Raskind et al.'s findings in a large randomized control trial that showed no significant benefit of prazosin over placebo [5]. This led to the removal or downgrade of prazosin from recommended treatments in many guidelines including the American Academy of Sleep Medicine [4, 6]. However, meta-analyses that include the Raskind trial have still shown a statistically significant benefit of prazosin in the treatment of PTSD-related nightmares [5–7].

The effects of psychotherapy and psychopharmacology are not immediate, and therefore, in the acute state, episodes of hypervigilance, paranoia, and flashbacks require more urgency in symptom alleviation to obtain stabilization. Literature reveals the ability of prazosin to decrease nightmares experienced with PTSD via the blockade of the alpha-1-receptor, leading to a downregulation of the responsible hypernoradrenergic response [8, 9]. Flashbacks, similarly to nightmares, have been linked to central nervous system adrenergic hyperarousal [10]. There is a lack of recent research and consistent findings, supporting the efficacy of valproic acid in the treatment of PTSD. While meta-analyses and retrospective reviews have shown efficacy in the reduction of symptoms of hyperarousal, intrusive thoughts, anxiety, and depression, the included studies had significant limitations [11, 12]. A randomized controlled trial studying the use of valproic acid in PTSD failed to show significant benefit over the placebo. However, the study only included a limited number of male veterans most of whom were combat-veterans with treatment-resistant PTSD [13]. In all these cases, studies were small or limited in their generalizability leaving a strong need for continued research in this topic, especially outside the combat-veteran population [12]. Recent literature on histone deacetylases in the pathophysiology of PTSD and valproic acid's role as a histone deacetylase inhibitor (HDACi) has brought a new light to the possible benefits of its role in PTSD treatment [14]. This case presentation focuses on the success of twice daily prazosin dosage in the treatment of PTSD. The utilization of a morning dose of prazosin is not widely reported on in literature but showed great promise in combination with valproic acid in the case presented.

## 2. Case Presentation

The patient was a 28-year-old, single, African-American, nulliparous female patient with a history of hypothyroidism (well controlled with levothyroxine), who presented involuntarily to the acute care inpatient psychiatry unit for paranoia and consistent visual hallucinations experienced randomly throughout the day over the past six months. The patient had earlier experienced several psychiatric hospitalizations since symptom onset, with reports of hyperarousal, paranoia, nightmares, and visual hallucinations composed of “writing on the walls.” The patient described words like “rape” and “die” being written in blood right before her eyes. The patient did not indicate a specific stressor at this time. The patient was clinically diagnosed with psychotic disorder, unspecified and subsequently started on risperidone 1 mg twice daily and prazosin for nightmare alleviation. During hospitalization, risperidone was titrated up to 3 mg twice daily and prazosin to 2 mg nightly, with reported nightmare alleviation, but minimum improvement of paranoia, hyperarousal, and visual hallucinations.

Upon further investigation, the patient disclosed a traumatic event occurring prior to symptom onset six months earlier and collateral information confirmed that the patient did not have any prior psychiatric history. The patient reluctantly disclosed a single episode of being violently raped at knife-point, with reports of laceration injury. The patient was ultimately diagnosed with PTSD following a structured interview in which the patient was found to fit the diagnostic criteria outlined in the DSM-5 according to the Clinician Administered PTSD Scale (CAPS-5) [15]. Risperidone was titrated down with eventual tapering off to cessation. Valproic acid 500 mg twice daily was initiated for the reduction of hyperarousal. An additional 2 mg dose of prazosin was added in the morning for the alleviation of the “visual hallucinations,” which were now deemed to be psychological responses (or intrusive symptoms of flashbacks) to the inciting event. After a prolonged hospitalization, these medication changes provided immediate and drastic improvement. The patient stabilized, and two days after the complete tapering of risperidone and initiation of the new medication regimen, the patient was able to be discharged from the acute care psychiatric unit.

## 3. Plan

The medical regimen for this patient included the following:

1. 500 mg valproic acid twice daily for arousal and reactivity symptoms of hyperarousal
2. 2 mg of prazosin in the morning for intrusive symptoms of flashbacks
3. 1 mg of prazosin at bedtime, for intrusive symptoms of nightmares

During the entirety of the hospitalization, the patient was closely monitored for efficacy and potential adverse effects of the medication regimen. This monitoring involved screening the patient multiple times a day for known adverse drug effects and evaluating the persistence and tracking the severity of the patient's flashbacks and hyperarousal. Nursing staff, social workers, residents, and attending physicians were all consulted daily and nightly in this monitoring process. Furthermore, the patient was offered supportive therapy and was eventually discharged with an outpatient psychiatry appointment and recommendations for continued trauma-focused cognitive behavioral therapy.

## 4. Discussion

This case presents a novel approach to the treatment of PTSD in a patient experiencing debilitating flashbacks and severe hyperarousal, which initially presented as a psychotic disorder. The patient was treated with a morning dose of prazosin in addition to her nighttime dose on the theory that her daytime flashbacks were similar in pathophysiology to her nightmares.

Valproic acid has been shown to improve hypervigilant symptoms and hyperarousal states associated with PTSD in some small, low-power studies [12]. There is a significant lack of recent research on the efficacy of valproic acid in the treatment of PTSD. Valproic acid's mechanism of action has not been fully elucidated; however, it has been shown to modulate serotonin pathways, dopamine pathways, and proinflammatory cytokines—all of which have been shown to play a role in the pathophysiology of PTSD [16–23]. One recent study has linked this modulation, in part, to its role as a HDACi and action in the serotonergic pathways [22]. Valproic acid's HDACi abilities have been of interest for more than just neurotransmitter modulation as the role of histone deacetylases in the pathophysiology of PTSD is investigated further [14]. Developing evidence is showing that histone deacetylases play a key part in PTSD from both an epigenetics and fear extinction perspective, which can possibly be treated with the HDACi mechanism of valproic acid leading to more efficient fear extinction [23–27].

Prazosin is an alpha-1-adrenergic receptor blocker typically used in the treatment of hypertension and benign prostatic hypertrophy [28]. Prazosin is commonly used in the treatment of nightmares and sleep improvement in PTSD, with its well-documented efficacy, which is linked to its ability to cross the blood-brain barrier [8, 29–32]. As previously discussed, there has been some debate on the benefits of prazosin, but the most recent meta-analyses still show significant efficacy for the treatment of nightmares [5–7]. This case presentation exhibited resolution of nightmares when treated with 1 mg of prazosin at bedtime. It is theorized that prazosin provides relief from nightmares due to its ability to downregulate the noradrenergic system [6, 33, 34]. PTSD sleep disturbances have been linked to upregulation of noradrenergic signaling during sleep, leading to decreased sleep efficiency, increased nocturnal awakenings, and increased duration of the rapid-eye-movement (REM) phase of sleep [35, 36].

In addition to nightmares, this patient was experiencing significant distress due to daytime hyperarousal and visual “hallucinations.” After the diagnosis of PTSD was made, the visual “hallucinations” were further explored and found to reference the traumatic event that was experienced by the patient. The authors postulated that due to similar symptomatology to nightmares, including signs of noradrenergic hyperarousal and references to the patient's trauma, these visual hallucinations could be comparable to “daytime nightmares” or flashbacks. Flashbacks are an individual form of memory described by Brewin as “situationally accessible” which references a traumatic event and depend far more on visuospatial processing than standard autobiographical memory [37, 38]. Furthermore, flashbacks have been found to be immersive and include more visual, motor, and emotional components, which could be explained by their correlation to adrenergic hyperarousal in the central nervous system [39] [10]. Given this patient's excellent response to treatment with prazosin for nightmares and the similarities discussed between this patient's nightmares and flashbacks, the patient was started on an additional morning dose of prazosin. Following this adjustment, a significant reduction in intrusive flashback symptoms, as well as concurrent noradrenergic symptoms, was noted.

Daytime dosing of prazosin has not been extensively researched, with only one double-blind study and three other documented cases found in our literature review [40, 41]. In fact, the very first Vietnam veteran treated with prazosin for PTSD included daily dosing of prazosin. Despite this early documentation, literature remains limited in this area. This first patient had severe nightmares and sleep disturbances following a traumatic event in Vietnam War in 1968 and was prescribed prazosin after failing a course of propranolol with intentions of somatic symptom alleviation. Prazosin resolved the nightmares, but the patient continued to have hyperarousal symptoms in the afternoon. A mid-morning and mid-afternoon dose of prazosin was then added to the veteran's medication regimen, and significant improvement was observed and sustained [41]. Decades later, a similar finding was observed in a study on the effectiveness of nighttime dosing of prazosin. Many participants continued to have daytime symptoms despite alleviation of nightmares with a bedtime dose of prazosin. This led to a subsequent study with the same cohort of participants which found that an additional morning dose of prazosin significantly decreased psychological distress in response to verbal trauma cues on emotional Stroop testing [40].

As discussed, flashbacks are a form of memory triggered by a patient's situation and are linked to noradrenergic hyperarousal. The authors postulate that prazosin was able to reduce flashbacks in this case presentation due to its ability to reduce both the psychological response to traumatic cues that could lead to flashback and the noradrenergic hyperarousal linked to flashbacks. While few studies indicate the success of daily prazosin dosing in PTSD, more studies are necessary to further expand upon our knowledge and understanding. Furthermore, future investigation is indicated to analyze the exact mechanism of action responsible for the reduction of the specifically intrusive flashbacks [33, 40].

Proper diagnosis with the appropriate treatment is crucial to a patient's prognosis. The original misdiagnosis of a psychotic disorder in this case presentation led to initial treatment with the use of an antipsychotic, which has not been recommended for the treatment of PTSD [4]. Particularly, in an acute setting, this can lead to prolonged suffering due to inadequate alleviation of symptoms. The authors stress the importance of a proper and prompt diagnosis of PTSD and suggest the use of tools, such as the PTSD Checklist civilian version (sensitivity ranged from 1 to 0.60 and specificity ranged 0.92 to 0.99) or other instruments based on the DSM, which showed an average efficiency of 0.85 [42]. After establishing a diagnosis of PTSD in this case presentation, the authors opted to implement treatment utilizing mood stabilization via histone deacetylate inhibition and noradrenergic modulation.

This patient suffered a prolonged state of psychological distress and failed many medication trials. The patient then showed significant improvement with prazosin and valproic acid treatment, but consideration must still be given to the use of teratogenic medications in females of reproductive age. Valproic acid is highly associated with neural tube defects and even increased rates of autism spectrum disorder in children exposed in utero; therefore, extensive patient education must be provided [43, 44]. Given this patient's severe distress prior to treatment, the patient and treatment team agreed that the benefits of treatment outweighed the risks. However, the patient was encouraged to start birth control and use barrier protection.

This case report displayed a strong and rapid correction of longstanding visual hallucinations and hyperarousal in a patient with PTSD. The authors utilized a physiological approach in choosing these medications to treat the underlying biologic cause of the patient's unique symptomatology. The particular strengths of this report lie within the patient's obvious clinical improvement displayed throughout the treatment process as well as her lack of relapse, or rehospitalization, as far as the authors are aware of. Assessment of the long-term efficacy of this treatment regimen was limited due to loss of follow-up with the patient's transition to outpatient care with a different psychiatric team. However, upon request for consent to publish this report, the patient was maintaining stability and doing well in the outpatient setting. Further limitations include the lack of clinical trials studying the efficacy and benefits of treatment with morning dosing of prazosin and incongruent research on the benefits of valproic acid.

## 5. Conclusion

This case presentation demonstrates successful treatment of the patient's intrusive flashbacks via twice daily prazosin and valproic acid. It is postulated that the shared pathophysiology of flashbacks and nightmares allows a similar treatment modality via noradrenergic downregulation with prazosin. Current research is lacking in the use of daytime prazosin and valproic acid's role as a HDACi in the treatment of PTSD. Further investigation is indicated and could lead to better clinical guidelines for the treatment of PTSD and ultimately a better prognosis, particularly in the acute setting.
